# How does on-demand delivery impact the accessibility and equity of community pharmacies? Perspective from groups with heterogeneous geographic-digital barriers

**DOI:** 10.3389/fpubh.2026.1872047

**Published:** 2026-07-29

**Authors:** Jing He, Shuyi Cao, Ningjun Ma, Xiaoyan Shi, Rui Wang, He Zhang

**Affiliations:** 1 Department of Architecture, Tianjin University, Tianjin, China; 2 Didi Chuxing, Beijing, China

**Keywords:** community pharmacy service, digital divide, equity, on-demand delivery, perception, potential accessibility

## Abstract

Accessibility to community pharmacy service (CPS) is a key concern in public health and spatial planning. Although on-demand delivery may expand service opportunities through digital platforms and third-party riders, its contribution to CPS potential accessibility and equity remains underexplored. Using 647 community units in central Tianjin, China, and survey-derived parameters from 600 questionnaires (83.5% response rate), this study extends the Two-Step Floating Catchment Area (2SFCA) method by incorporating multidimensional supply capacity and a time-decay function weighted by modeled supplier-selection probability. Communities were classified into four groups according to geographic and digital barriers, and Geodetector was applied to examine factors associated with spatial heterogeneity in potential accessibility. Results reveal substantial group differences. In-store dispensing provides higher potential accessibility for groups with low geographic barriers, whereas on-demand delivery shows a relative accessibility advantage for groups facing high geographic or dual barriers. For these groups, delivery is also associated with a more equitable distribution of potential service opportunities, with Gini coefficients 0.08-0.12 lower than those for in-store dispensing. Factor interactions generally show greater explanatory power than individual factors, with prominent associations shifting from population size to interactions involving age structure and socioeconomic characteristics. These findings support differentiated pharmacy planning and closer coordination between offline mobility and online delivery networks.

## 1 Introduction

Global healthcare expenditure continues to rise due to population aging, burden of chronic disease, and advances in medical technology (1). As a core component of primary healthcare, community pharmacy service (CPS) ensures timely access to medications through spatial proximity and appointment-free provision (2, 3). CPS scope varies internationally, with basic services like dispensing common, while regions such as Europe and the U.S. have expanded to disease management, vaccination, and health promotion (4, 5). For people hindered by physical distance, mobility, or other barriers, CPS may constitute an essential or even sole source of healthcare support (6, 7). To promote Sustainable Development Goals of healthy lives for all ages (8), assessing the potential accessibility and equity of CPS, has therefore become a key issue across public health, spatial planning, and transportation.

The COVID-19 pandemic and diffusion of Information and Communications Technology (ICT) have accelerated the global shift from in-store dispensing to pharmaceutical e-commerce (9–11), with its global market reached USD 234.95 billion in 2025, led by America in share (39.71%) and Asia-Pacific region in growth (13.12%) (12). In China, policies like simplified electronic prescription rules, and infrastructures like front warehouses, have made on-demand delivery the dominant e-commerce mode (10). Leveraging digital platforms and delivery riders, this channel provides order management and hourly delivery for partner pharmacies. Given that on-demand delivery retains core functions of in-store dispensing and therefore has the potential to complement or replace this traditional mode, it is vital to examine whether it offers an advantage in potential accessibility.

Regarding the above issues, studies have predominantly focused on the impacts of on-demand delivery within food service (9, 13, 14). For instance, Zhang et al. (14) found that on-demand delivery overall improved food accessibility, notably remedying the accessibility deficits of low-mobility transport modes including walking and public transit, while substantially enhancing food accessibility in suburban and rural communities. In terms of equity, it widened urban–rural spatial disparities, and vulnerable groups such as the older population encountered barriers to using on-demand delivery. Focusing on South Korea, Kim et al. (15) revealed that on-demand delivery exacerbated rather than alleviated spatial inequality in food access; their study further identified an accessibility threshold effect among low-income groups and a significant negative correlation between the older population and comprehensive food accessibility.

While these studies have centered on individual and urban–rural disparities, overlooking structural barriers inherent to service channels difficult for individuals to avoid (16, 17). Grounded in cognitive process theory (18–20), individuals face distinct constraints when choosing service channels: in-store dispensing is primarily constrained by geographic barriers (21), manifested as disparities in travel distance and mobility costs due to uneven spatial distribution (Roubin et al. (22)); in contrast, on-demand delivery is constrained by digital barriers, where divides in broadband access and digital literacy exclude some ethnic minorities and low-income groups (17, 23). However, prior studies often analyze these barriers in isolation, lacking a unified framework that integrates geographic and digital constraints. Consequently, a critical gap remains in identifying the demand groups, those facing geographic barriers who are also best positioned to potentially benefit from on-demand delivery.

Second, on-demand delivery has reshaped the core dimensions of potential accessibility (24). Existing studies, however, primarily quantify it based on objective spatial conditions. For example, Kim et al. (15) measured binary food accessibility, namely the mere presence of establishments providing on-demand delivery. Kong et al. (10) applied the cumulative opportunity method to CPS by counting suppliers within a specified delivery radius. Three key limitations exist: (1) The distance decay effect inherent to delivery channel, particularly users’ sensitivity to timeliness, is ignored; (2) Critical factors including demand scale, user preferences, and supply competition constrained by rider capacity and concurrent demand at peak period are overlooked, resulting in inconsistencies with real-world supply–demand interactions (25); (3) Lack of pharmaceutical service specificity limits their practical relevance for CPS optimization.

Finally, identifying the factors associated with potential accessibility heterogeneity is essential for optimizing service provision. While existing studies have examined socioeconomic and spatial influences on healthcare accessibility (2, 17, 26–28), they focus predominantly on the independent effects of single factors, with insufficient attention to interaction effects like synergy or antagonism. Since CPS accessibility is a comprehensive outcome associated with multi-factor interactions, single-factor analyses cannot fully explain the observed disparities. Exploring these interactive effects better reflects the complexity of driving mechanism and is essential for developing effective policy interventions.

To address these limitations, namely the lack of integrated identification of channel-specific barriers, behaviorally informed measurement of potential accessibility, and analysis of factor interactions, this study examines the potential contribution of on-demand delivery to CPS accessibility through three questions: (1) How can objective spatial conditions and survey-derived perceptual parameters be integrated to measure potential CPS accessibility? (2) Across groups facing different geographic and digital barriers, does on-demand delivery provide higher potential accessibility and a more equitable distribution of potential service opportunities than in-store dispensing? (3) Is the spatial heterogeneity of potential accessibility explained primarily by individual factors or by their interactions?

To answer these questions, this study analyzes 647 community units in Tianjin, a megacity in northern China: (1) The Two-step floating catchment area (2SFCA) model is extended to quantify potential CPS accessibility through both in-store dispensing and on-demand delivery. Based on survey-derived parameters concerning residents’ preferences, weights of supply capacities and a time-decay function weighted by supplier-selection probability are incorporated; (2) Community units are classified into groups with distinct geographic and digital barriers based on in-store supplier coverage and digital penetration, and the potential accessibility and spatial equity of the two channels are compared across groups; (3) For groups in which on-demand delivery shows a potential accessibility advantage over in-store dispensing, Geodetector is applied to examine the individual and interaction explanatory power of factors associated with spatial heterogeneity in potential accessibility.

**Figure 1:**
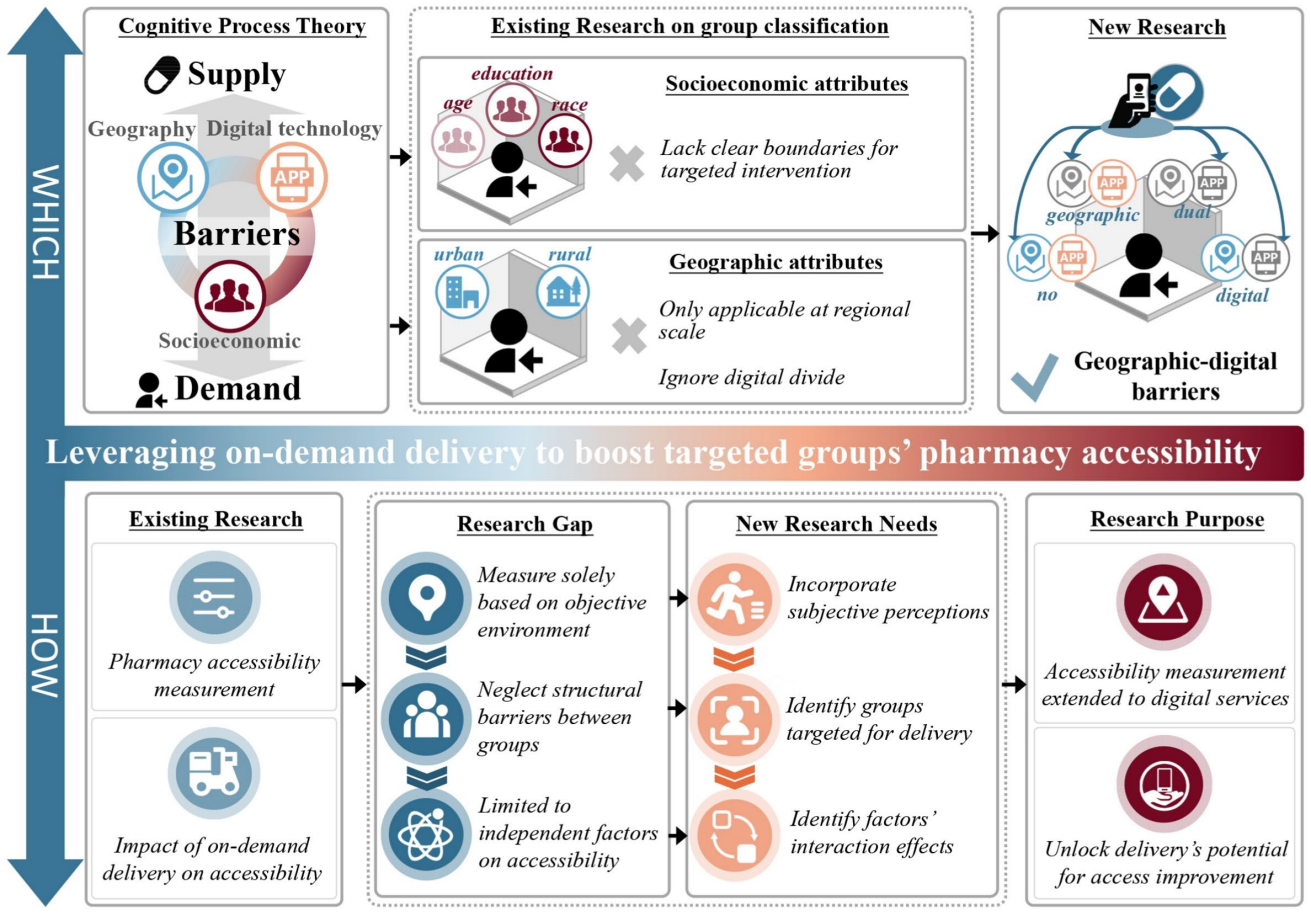
Research conceptual framework.

Theoretically, this study extends the 2SFCA framework by integrating objective spatial conditions with survey-derived perceptual parameters, enabling the measurement of potential CPS accessibility across physical and digital service channels. Practically, it identifies barrier-specific groups for which on-demand delivery offers the greatest modeled accessibility advantage and reveals the factors associated with spatial heterogeneity in potential accessibility. The findings provide planning-oriented evidence for differentiated pharmacy-network development and digital health policy (Figure 1).

## 2 Literature review

### 2.1 Differences between in-store and delivery-based accessibility

Advances in ICT has facilitated the global expansion of on-demand delivery, enabling online access to routine medications through digital platforms and delivery riders (10). By reducing the need for individual travel, this model reshapes the land-use, transport, and individual dimensions of potential accessibility: it extends service areas beyond walkable neighborhoods, replaces short-distance trips with delivery services, and shifts user considerations from proximity toward delivery time, cost, and store ratings (9, 13, 29, 30). However, existing assessments of CPS have insufficiently considered differences in service utility and participation barriers between channels, potentially limiting their representation of residents’ potential access to medications.

Service equivalence is a prerequisite for comparing the potential accessibility of on-demand and in-store CPS. Unlike telemedicine, which cannot fully replace physical diagnosis and examinations and therefore mainly complements in-person care (31–33), on-demand delivery can preserve key pharmacy functions such as consultation, product selection, and receipt while removing face-to-face interaction (16). Similar substitution has been observed in food and retail services, supporting the potential of delivery to extend service accessibility (14, 15).

However, realizing this potential depends on participation in digital services. Digital barriers arise from both device and network access and the literacy required to use online platforms (33). Despite widespread digital infrastructure, inequalities in service quality and digital skills persist, particularly among older adults (21, 23). Thus, evaluating on-demand delivery requires jointly identifying the geographic and digital barriers faced by different demand groups; otherwise, its potential accessibility advantage may remain unrealized or reinforce existing disparities.

### 2.2 Potential accessibility integrating objective and perceived dimensions

Accessibility refers to the potential or ease of reaching desired opportunities (34), such as employment, goods, or services (35). It is commonly structured by land use, transport, individual, and temporal components (24). Traditional quantitative approaches primarily measure objective accessibility. Early methods assessed spatial proximity by counting opportunities within predefined distance or time thresholds, including network, buffer, and kernel-density methods (36, 37). The gravity model subsequently incorporated the decay of supply–demand interaction with distance (38), while the Two-Step Floating Catchment Area method restricted such interactions to predefined service areas (60). Owing to its transparent structure, 2SFCA has been widely extended by incorporating supplier-selection probabilities and multimodal transport conditions (61, 62).

However, objective measures alone may overlook differences in individual preferences and perceived ease of access. Perceived accessibility refers to individuals’ evaluations of whether opportunities can be reached conveniently (39) and is commonly measured through access-process evaluations (40, 41). For example, perceived accessibility has been assessed using the gap between actual and acceptable travel time or evaluations of walking conditions (25, 42). Nevertheless, purely perceptual measures are often weakly connected to the physical service environment, limiting their use in spatial intervention (20).

To retain an objective spatial basis while accounting for individual perceptions, recent studies have incorporated survey- or behavior-derived parameters into accessibility models (43–45). Healthcare trajectories have been used to calibrate catchment areas and distance-decay functions, while age-specific acceptable walking times have informed service radii in park-accessibility models (25, 46). These parameters improve the behavioral sensitivity of potential accessibility estimates but do not represent realized service utilization.

Existing parameters remain highly context-specific and rarely address pharmaceutical delivery. In particular, delivery-time tolerance and preferences regarding virtual suppliers have received limited attention. Incorporating channel-specific perceptual parameters into objective spatial models is therefore necessary to estimate the potential accessibility of CPS under both in-store and on-demand delivery conditions.

### 2.3 Factors associated with spatial heterogeneity in CPS accessibility

Spatial disparities in potential access to offline health services are associated with both spatial conditions, such as facility location and supply–demand scale, and non-spatial characteristics, including income and demographic structure. Existing studies have mainly examined general or multi-tier healthcare systems rather than pharmacies. Their findings suggest that facility density, population distribution, urbanization, income, and walking-network conditions are associated with spatial variation in healthcare accessibility, although the strength and direction of these associations differ across facility types (26–28).

Evidence regarding digital pharmaceutical services remains limited. Studies of telemedicine and delivery services indicate that broadband access, area deprivation, urban–rural location, housing prices, population density, and land-use intensity are associated with online service availability and accessibility (10, 17).

However, most studies assess factors independently and pay limited attention to their interactions. Because spatial and socioeconomic conditions may jointly explain disparities in potential accessibility, examining both individual and interaction explanatory power is necessary to better characterize its spatial heterogeneity.

## 3 Materials and methods

### 3.1 Study area

**Figure 2:**
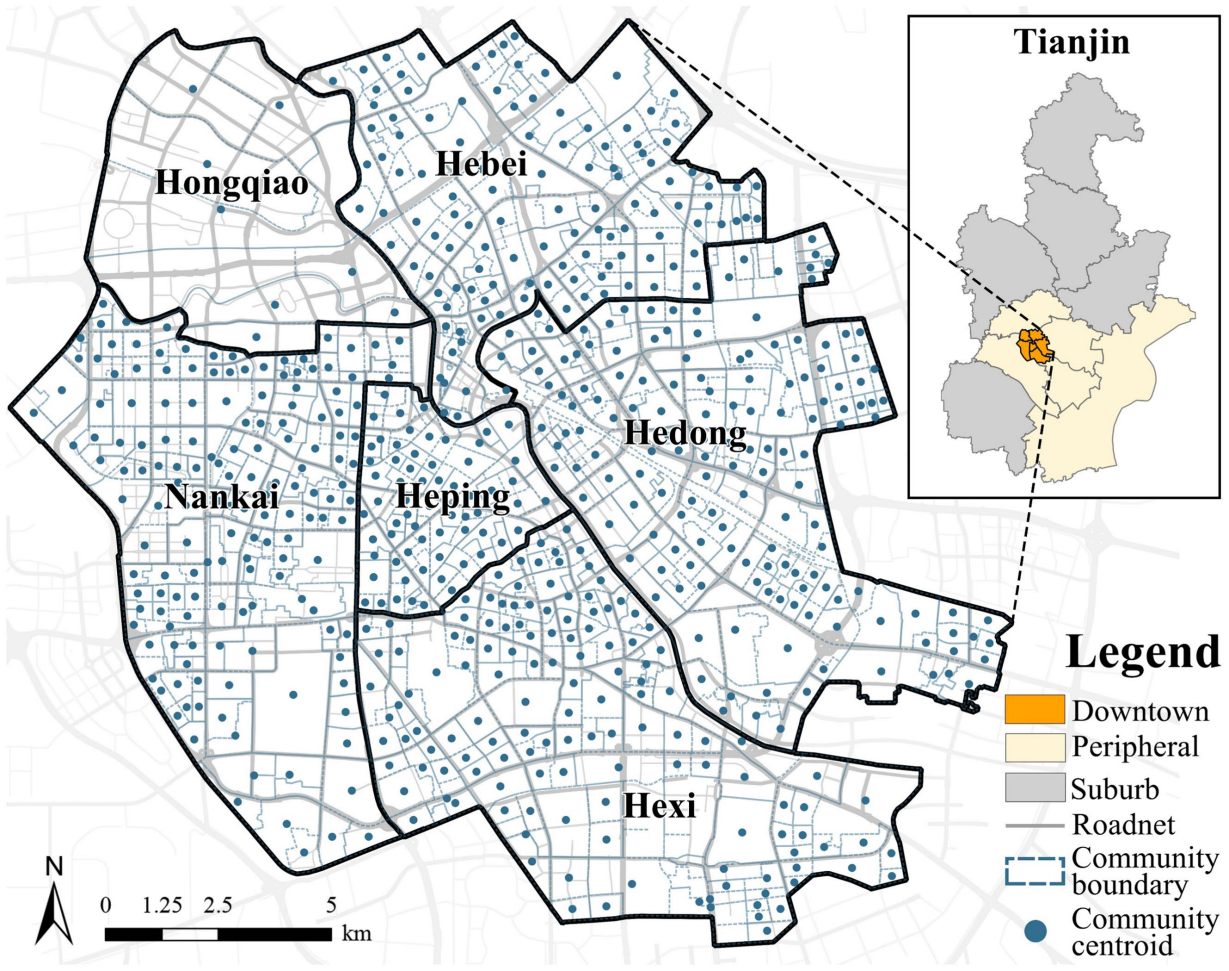
Study area.

This study focuses on the urban area of Tianjin, as shown in Figure 2. Tianjin recorded 5,830 pharmaceutical enterprises in 2024, surpassing Beijing and Shanghai (47). On the demand side, population density of each district in the study area is roughly 10 times that of outer suburbs and rural areas. Meanwhile, 22% of the population are aged 60 and above, indicating a moderately aging society. And the aging rate in the research area is higher than the citywide average, with Hebei District and Hongqiao reaching 31% (48).

Besides, this study adopts 647 communities under subdistrict jurisdiction as the analysis units. In China’s grassroots governance, a “community” refers to the geographic scope where neighborhood committees provide public services and conduct management. Compared with homogeneous grids, the community-based unit offers distinct advantages: (1) it aligns closely with the spatial coverage of CPS, facilitating policy evaluation and intervention; (2) it integrates key attributes such as population and socioeconomic characteristics, avoiding internal heterogeneities that may be masked by uniform grid zoning.

### 3.2 Data collection

Table 1 summarizes the data sources. In-store suppliers comprise Primary Healthcare Institutions (PHIs), including community hospitals, outpatient departments, and community health service stations, and Pharmaceutical Outlets (POs) defined in the community living-circle standard (49, 59). Data on on-demand suppliers were collected from Meituan, a leading delivery platform in China. Using the centroid of each community unit as a simulated delivery address, we systematically recorded all suppliers and service attributes displayed as available to that unit.

**Table 1:** Summary of data sources.

Type	Description	Source	Time
In-store suppliers	Point-of-Interest (POI) of PHI and PO, including names and geographic coordinates.	*Amap*	2023
	Name, business hours of the above POIs.	*Dianping*	2023
	Walking time from each community centroid to the corresponding POI.	*Amap* API	2023.12
	List of “designated retail pharmacies for medical insurance.”	Tianjin Municipal Healthcare Security Bureau	2023.12
On-demand delivery suppliers	List of “Internet hospital.”		2023.12
	Suppliers displayed in the “*Medical Treatment & Medicine Purchase*” section, including ratings, delivery options and fees, minimum order prices, coupons, and platform rankings.	*Meituan*	2023.10
Pharmacy consumption behavior	Residents’ stated preferences and acceptable access thresholds for potential accessibility modeling.	Questionnaire	2023.10
Service facilities	POI of general hospitals and commercial facilities, including names and geographic coordinates.	*Amap*	2023
Cell tower	Identification code, network standards (2G/3G/4G/5G), and geographic coordinates.	*OpenCelliD*	2023
Demographic attributes	Street-level demographic data, including total population and age structure.	Seventh National Population Census of China	2020

**Figure 3:**
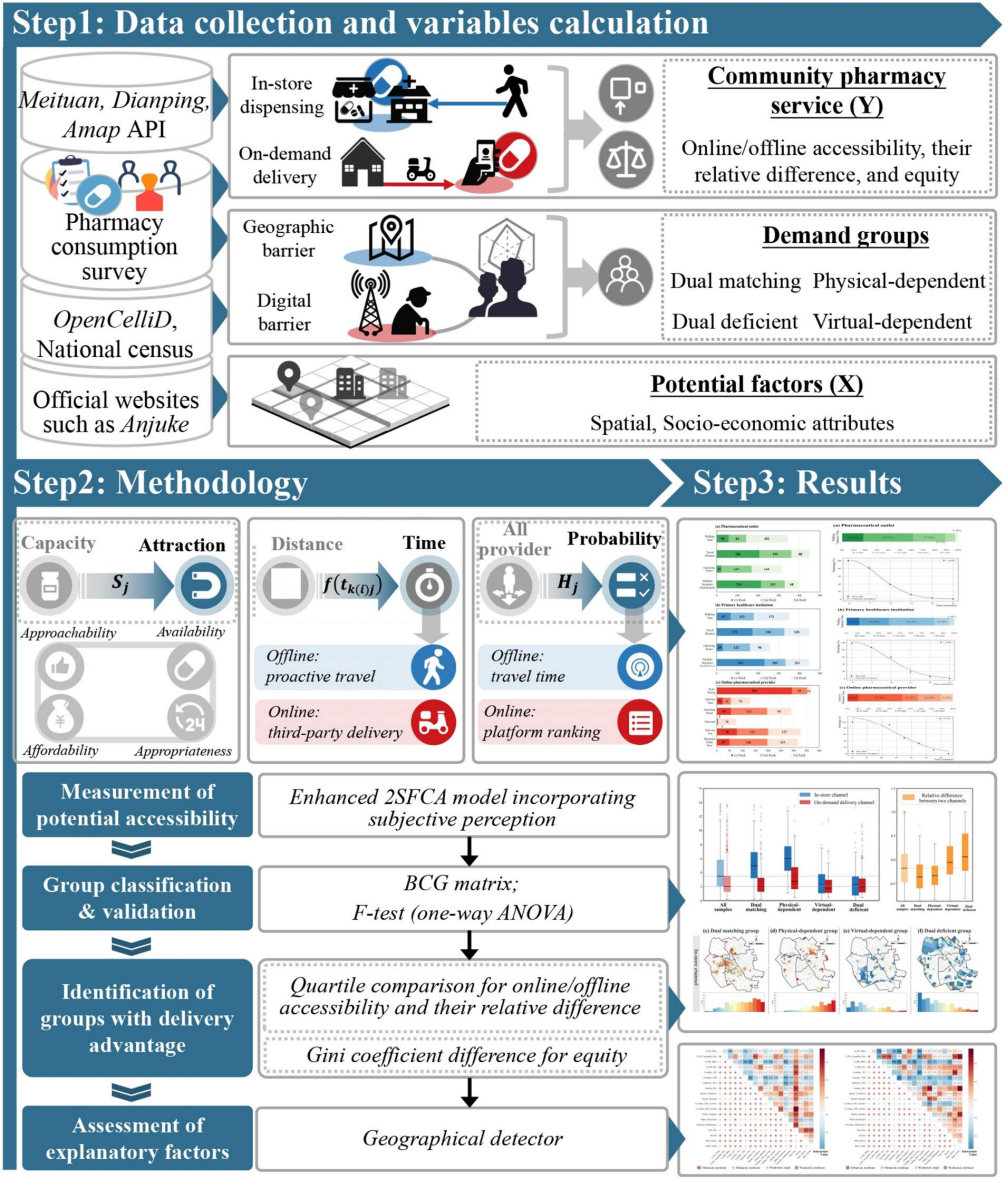
Research workflow.

On the demand side, a stratified questionnaire survey was conducted to derive stated preferences and acceptable access thresholds for calibrating the potential accessibility model. Sampling followed Tianjin’s population age structure. Of the 600 questionnaires distributed, 501 were valid, yielding an effective response rate of 83.50%. The sampling protocol and questionnaire are provided in Supplementary Notes 1–2. Figure 3 presents the research workflow.

### 3.3 Quantification of potential accessibility and equity

#### 3.3.1 Refinement 1: multidimensional supply capacity of CPS

Supplier capacity refers to its relative attractiveness in residents’ decision-making. Existing studies often use facility size, staffing, or equipment quantity to measure supply capacity (50), but these indicators are less suitable for CPS because drug portfolios are already highly standardized. Residents’ choices depend on whether a supplier is visible, convenient, affordable, and temporally compatible with their needs. Accordingly, this study uses the preference-weighted multidimensional indicators in Table 2 as proxies for supply capacity (51).

**Table 2:** Indicators and weights of supply capacity.

Supply capacity	On-demand delivery		In-store dispensing		
	Indicator	w	Indicator	$w_{\mathrm{PHI}}$	$w_{\mathrm{PO}}$
Approachability	Store rating	0.24	-		
Availability	*Meituan-*dedicated delivery^1^ (Yes = 1, No = 0)	0.09	Spatial proximity	0.30	0.30
			Walking convenience	0.23	0.23
Affordability	Minimum order price	0.21	Medical insurance qualification (Yes = 1, No = 0)	0.30	0.26
	Delivery fee	0.22			
	Coupon number	0.05			
Appropriateness	24-h operation (Yes = 1, No = 0)	0.19	24-h operation (Yes = 1, No = 0)	0.17	0.21

^1^This mode refers to a delivery service carried out by dedicated couriers employed by *Meituan*. Orders are proactively assigned by dispatch stations and cannot be rejected by couriers, ensuring stable and timely delivery.

These indicators are channel-specific because in-store dispensing and on-demand delivery operate through different service mechanisms: (1) in the delivery channel, approachability is mainly driven by platform information, so platform ratings are used to capture supplier visibility and recognizability; no comparable measure is set for the in-store channel because review data are unavailable. (2) In the delivery channel, availability reflects platform-based service capacity, while in the in-store channel it reflects spatial convenience, measured by each supplier’s average distance and walking time to community units. (3) In the delivery channel, affordability is mainly shaped by minimum order price, delivery fees, and coupons; in the in-store channel, it is determined by insurance-payment eligibility, which directly affects residents’ out-of-pocket costs and was not available for online use in Tianjin in 2023 (52). (4) Appropriateness is defined consistently across both channels as the alignment between operating hours and residents’ temporal needs.

Survey respondents ranked the importance of the indicators (Supplementary Note 3), each of m supply dimension is assigned a weight $w_{m}$ according to the frequency with which residents rank it as the top priority. For any supplier *j*, the revised supply capacity $S_{j}$ is computed as the weighted sum of its normalized indicator scores $S_{\mathrm{jm}}$ (Equation 1). PO and PHI capacities are calculated separately and then aggregated using survey-reported supplier-type selection shares of 0.48 and 0.52.


$$S_{j}=\sum_{m=1}^{M}w_{m}\cdot S_{\mathrm{jm}}$$ (1)

#### 3.3.2 Refinement 2: time-decay function weighted by supplier-selection probability

Time decay function (Equation 2): To capture the decline in potential accessibility as impedance increases, this study uses time as the impedance variable linking CPS suppliers and users. Compared with Euclidean or network-distance measures (15, 53), time better reflects service acquisition costs, with walking time used for in-store dispensing and delivery time for on-demand delivery. Both the time threshold and functional form are fitted to residents’ self-reported maximum acceptable time in the questionnaire (Supplementary Note 4).


$$f(t_{k(i)j})=\{\begin{array}{c} g(t_{k(i)j})\cdot H_{j},\;\;\;t_{k(i)j}\leq t_{0}\\ 0,\;\;\;t_{k(i)j}>t_{0} \end{array}$$ (2)

Where $t_{k(i)j}$ denotes interaction time between the demander k(i) and supplier *j*, and $t_{0}$ is the time threshold.

Supplier-selection probability (Equation 3): Traditional models may overestimate potential demand by assuming users access all reachable suppliers equally. Drawing on the Huff model, this study introduces a supplier-selection probability to represent competition among suppliers within each catchment area (65). For in-store dispensing, competition is proxied by travel time (45, 46); for on-demand delivery, platform ranking is used to reflect differences in supplier visibility and competitiveness (54). The resulting probability $H_{j}$ is incorporated into the time-decay function:


$$H_{j}=\frac{S_{j}h(x_{j})}{\sum_{m=1}^{n}S_{m}h(x_{m})}$$ (3)

where *m* denotes a competing supplier within the catchment area of community *i*; n is the number of competing suppliers; $S_{j}\;$ is the supply capacity of supplier *j*; $h(x_{j})$ denotes the decay function of the corresponding competitive variable *x*, with channel-specific functions fitted as described in Supplementary Note 5.

#### 3.3.3 Potential accessibility across two channels and their relative difference

The formulas for calculating potential accessibility of in-store dispensing ( $PA_{i}$ ) at unit *i* are as follows (Equations 4, 5):


$$\begin{array}{l} PA_{i}=w_{\mathrm{PHI}}\cdot \sum_{j\in \{t_{\mathrm{ij}}\leq t_{0}^{\mathrm{PHI}}\}}\frac{S_{j}^{\mathrm{PHI}}g(t_{\mathrm{ij}})H_{j}^{\mathrm{PHI}}}{\sum_{k\in \{t_{\mathrm{kj}}\leq t_{0}^{\mathrm{PHI}}\}}D_{k}g(t_{\mathrm{kj}})}\\ +w_{\mathrm{PO}}\cdot \sum_{j\in \{t_{\mathrm{ij}}\leq t_{0}^{\mathrm{PO}}\}}\frac{S_{j}^{\mathrm{PO}}g(t_{\mathrm{ij}})H_{j}^{\mathrm{PO}}}{\sum_{k\in \{t_{\mathrm{kj}}\leq t_{0}^{\mathrm{PO}}\}}D_{k}g(t_{\mathrm{kj}})} \end{array}$$ (4)

$$H_{j}^{\mathrm{PHI}/\mathrm{PO}}=\frac{S_{j}^{\mathrm{PHI}/\mathrm{PO}}h(t_{j})}{\sum_{m\in \{t_{\mathrm{im}}\leq t_{0}^{\mathrm{PHI}/\mathrm{PO}}\}}S_{m}^{\mathrm{PHI}/\mathrm{PO}}h(t_{m})}$$ (5)

Where $w_{\mathrm{PHI}}$ , $w_{\mathrm{PO}}$ are the preference weights for PHI and PO, respectively; $S_{j}^{\mathrm{PHI}/\mathrm{PO}}$ represents the supply capacity of supplier *j*; $D_{k}$ is the demand scale of unit *k*; g(t) is a travel-time decay function with $t_{0}^{P}$ as the threshold; $H_{j}^{\mathrm{PHI}/\mathrm{PO}}$ is the probability of supplier *j* being selected; $h(t_{j})$ is the decay function based on the travel time to supplier *j*.

Likewise, potential accessibility of on-demand delivery ( $VA_{i}$ ) are calculated as follows (Equations 6, 7):


$$VA_{i}=\sum_{j\in \{t_{\mathrm{ij}}\leq t_{0}^{V}\}}[\frac{S_{j}^{V}g(t_{\mathrm{ij}})H_{j}^{V}}{\sum_{k\in \{t_{\mathrm{kj}}\leq t_{0}^{V}\}}D_{k}g(t_{\mathrm{kj}})}]$$ (6)

$$H_{j}^{V}=\frac{S_{j}^{V}h(r_{j})}{\sum_{m\in \{t_{im}\leq t_{0}^{V}\}}S_{m}^{V}h(r_{m})}$$ (7)

Where $S_{j}^{V}$ represents the supply capacity of supplier *j*; g(t) is a delivery-time decay function with $t_{0}^{V}$ as the threshold; $H_{j}^{V}$ is the probability of supplier *j* being selected; $h(r_{j})$ is the decay function based on the platform ranking of supplier *j*.

To compare the relative potential accessibility provided by the two service channels in each community unit, this study calculates the relative accessibility difference $rA_{i}$ between on-demand delivery and in-store dispensing (Equation 8). This dimensionless index ranges from −1 to 1. A positive value indicates higher potential accessibility through on-demand delivery, whereas a negative value indicates an advantage for in-store dispensing. A larger absolute value $rA_{i}$ , represents a greater relative difference between the two channels. The index therefore identifies the channel offering potential accessibility advantage, rather than indicating realized service use or direct substitution between channels.


$$rA_{i}=\frac{VA_{i}-PA_{i}}{VA_{i}+PA_{i}}$$ (8)

#### 3.3.4 Equity of potential accessibility

To assess the equity of potential CPS opportunity distribution, this study uses the Lorenz curve and Gini coefficient. Community units are ranked by potential accessibility, with cumulative population share on the horizontal axis and cumulative potential accessibility share on the vertical axis. The 45° line represents perfect equality, while greater deviation indicates a more unequal distribution. The Gini coefficient is calculated as follows (Equation 9):


$$G=1-\sum_{i}^{n}(\mathrm{po}p_{i+1}-\mathrm{po}p_{i})(A_{i+1}+A_{i})$$ (9)

where *n* is the total number of units, $\mathrm{po}p_{i}$ is the cumulative population proportion up to the *i*-th unit sorted by accessibility, and $A_{i}$ is the corresponding cumulative proportion of potential accessibility. Gini coefficient ranges from 0 to 1, with higher values reflecting greater inequality in accessibility distribution.

### 3.4 Identification of groups with on-demand delivery advantage

#### 3.4.1 Geographic barrier

The spatial distribution of in-store suppliers is uneven due to policy, market, and other factors, resulting in significant disparities in the time, financial, and physical costs incurred by residents. To quantify the geographic barrier, this study uses the coverage rate of in-store suppliers $PB_{i}$ : a higher value indicates lower difficulty in traveling and thus a weaker geographic barrier.

The calculation follows three steps: (1) Kernel density estimation in ArcGIS is applied to compute the density ${\hat{f}}_{k}(m)$ of type-*k* in-store supplier at grid *m* (Equation 10). Bandwidth $h_{k}$ is set according to the service radius specified for such facilities in official standards (49); travel beyond this radius implies costs that exceed the service value. (2) Using zonal statistics in ArcGIS, grid-level density values are aggregated to unit *i* with area as the weight, yielding the per-unit-area coverage rate $PB_{i}^{k}$ of type-*k* supplier in unit *i* (Equation 11). (3) Coverage rates of all *q* types (here, PHI and PO) are summed to obtain $PB_{i}$ for unit *i* (Equation 12).


$${\hat{f}}_{k}(m)=\frac{1}{n_{k}h_{k}^{2}}\sum_{p=1}^{n_{k}}K(\frac{d_{\mathrm{mp}}}{h_{k}})$$ (10)

$$PB_{i}^{k}=\frac{1}{C_{i}}\sum_{m\in M_{i}}\frac{c_{i,m}}{C_{m}}\cdot f_{k}(m)$$ (11)

$$PB_{i}=\sum_{k=1}^{q}PB_{i}^{k}$$ (12)

Where $n_{k}$ is the total number of in-store suppliers of type *k*; $d_{\mathrm{mp}}$ is the Euclidean distance from the centroid of grid *m* to supplier *p*; K(⋅) is the kernel function, which assigns higher weights to shorter distances within the service radius and zero weight outside the radius; $c_{i,m}$ is the area of grid *m* that falls within community unit *i*; $C_{m}$ , $C_{i}$ are total areas of grid *m* and unit *i*, respectively; $M_{i}$ is the set of all grids intersecting with unit *i*.

#### 3.4.2 Digital barrier

For the delivery channel, this study quantifies the digital barrier using digital technology penetration rate $VB_{i}$ , constructed from two components: communication device access and digital literacy. A higher technology penetration rate indicates a lower barrier for residents in community unit *i* to use digital platforms.

Specifically: (1) Communication device access $L_{i}$ is measured by the weighted sum of the quantity of mobile communication bases and their corresponding network transmission rates within unit *i* (Equation 13). This study covers 2G to 5G bases, which follow the trend of shrinking coverage radius (10 km to 0.5 km) but rising transmission rate (150 Kbps to 100 Mbps) across successive generations. (2) Digital literacy $p_{i}$ refers to the ability to use online information and digital platforms effectively. Given limited community-level data for the full population, this study uses the proportion of older adults able to use digital platforms μ based on questionnaire as a proxy, as they are more vulnerable to digital exclusion (Equation 14). Technology penetration rate is defined as the product of the above two dimensions (Equation 15):


$$L_{i}=\frac{\sum_{b=1}^{s}N_{\mathrm{ib}}l_{b}}{C_{i}}$$ (13)

$$P_{i}=\frac{\rho_{i}}{\rho_{i}}\cdot r_{i}\cdot o_{i}\mu$$ (14)

$$VB_{i}=L_{i}\cdot p_{i}$$ (15)

Where $N_{\mathrm{ib}}$ is the number of type-*b* base in unit *i*; $l_{b}$ is the network transmission rate of type-*b* base; *s* is the total number of base types; $C_{i}$ is the area of unit *i*; $\rho_{i}$ is the population density of unit *i*; ${\bar{\rho }}_{i}$ is the average population density across the study area; $r_{i}$ is the proportion of residential land; $o_{i}$ is the aging rate of unit *i*.

#### 3.4.3 Demand group classification and validity assessment

After standardizing coverage rate of in-store suppliers $PB_{i}$ and digital technology penetration rate $VB_{i}$ , this study applies an adapted BCG matrix to classify community units by their geographic and digital barriers. Median values are used as thresholds to reduce sensitivity to outliers and skewed distributions while maintaining relatively balanced group sizes.

Table 3 presents four groups representing different combinations of the two barriers. Compared with clustering or dimensionality-reduction methods, this approach preserves the independent information of both indicators and makes their joint configuration directly interpretable.

**Table 3:** Demand group classification.

Demand group	Relationship between $PB_{i}$ and $VB_{i}$	Channel barriers
Dual matching	Both $PB_{i}$ and $VB_{i}$ are above the median.	Weak geographic and digital barriers
Physical-dependent	$PB_{i}$ above the median; $VB_{i}$ below the median.	Weak geographic barrier, strong digital barrier
Virtual-dependent	$VB_{i}$ above the median; $PB_{i}$ below the median.	Weak digital barrier, strong geographic barrier
Dual deficient	Both $PB_{i}$ and $VB_{i}$ are below the median.	Strong geographic and digital barriers

A one-way ANOVA was conducted to test whether the four barrier groups differ significantly in in-store potential accessibility, delivery-based potential accessibility, and their relative difference. The total variation $SS_{T}$ is decomposed into between-group variation $SS_{b}$ and within-group variation $SS_{w}$ , and the F-statistic is calculated as their variance ratio. A larger *F*-value indicates that between-group differences are greater relative to within-group variation. Statistical significance is assessed at the specified alpha level. The formulas are as follows (Equations 16–19):


$$SS_{T}=SS_{b}+SS_{w}$$ (16)

$$MS_{b}=\frac{SS_{b}}{df_{b}}=\frac{\sum_{i=1}^{k}n_{i}{\left({\bar{x}}_{i}-{\bar{x}}_{\text{total}}\right)}^{2}}{k-1}$$ (17)

$$MS_{w}=\frac{SS_{w}}{df_{w}}=\frac{\sum_{i=1}^{k}\sum_{j=1}^{n_{i}}{\left(x_{\mathrm{ij}}-{\bar{x}}_{i}\right)}^{2}}{N-k}$$ (18)

$$F=\frac{MS_{b}}{MS_{w}}\sim F(df_{b},df_{w}\;)$$ (19)

Where *k* is the number of barrier groups (*k* = 4); $n_{i}$ is the number of units in group *i*; $x_{\mathrm{ij}}$ is the potential accessibility of community unit *j* in group *i*; ${\bar{x}}_{\text{total}}$ , ${\bar{x}}_{i}$ are the overall and group-specific means; *N* is total number of units; $MS_{b}$ , $MS_{w}$ are the between- and within-group mean squares; $df_{b},df_{w}$ are the corresponding degrees of freedom.

#### 3.4.4 Comparative assessment of accessibility across groups

To compare the scale and equity of potential accessibility of CPS across demand groups, this study uses box plots and Gini coefficients, with the full sample as a reference.

First, in-store and delivery-based potential accessibility are compared within each group. Because both indicators are non-negative and right-skewed, they are log-transformed and standardized before comparison. Second, the relative accessibility difference is used to identify which channel provides a potential accessibility advantage, particularly for the virtual-dependent and dual-deficient groups facing high geographic barriers. Finally, channel-specific Gini coefficients are compared within each group to assess whether on-demand delivery is associated with a more equitable distribution of potential service opportunities than in-store dispensing.

### 3.5 Analysis of factors associated with CPS accessibility

#### 3.5.1 Selection of potential factors

To examine whether the factors associated with potential CPS accessibility differ between service channels, this study focuses on groups in which on-demand delivery shows a modeled accessibility advantage and a more equitable distribution of potential service opportunities. Potential explanatory factors are organized into three categories: spatial attributes of in-store dispensing and on-demand delivery, demand-side socioeconomic characteristics (Table 4).

**Table 4:** Summary of potential factors.

Factors	Variables	Description
In-store dispensing	N_PO_300m	Number of in-store suppliers within the maximum travel distance acceptable to residents and certain radius based on standards (49).
	N_PO_Acceptable_Dist	
	N_PHI_300m	
	N_PHI_1km	
	Centrality_PO	Average straight distance between all establishments and the unit’s geometric center (m).
	Centrality_PHI	
	Hierarchy_CPS	Level 0 (no PO and PHI), Level 1 (only PO or PHI), and Level 2 (both PO and PHI).
	Landuse_Mix	Shannon entropy of various land uses within each unit.
	Density_Commerce	Density of commercial facilities (or general hospitals^1^) within each unit (units/km^2^).
	Density_Hospital	
On-demand delivery	N_Online_CPS_AccTime	Number of online suppliers within the maximum delivery time or distance (3 km) acceptable to residents (58).
	N_Online_CPS_AccDist	
	Online_Penetration	Proportion of online suppliers to the total number of pharmacy establishments within the unit.
	Proximity_WebHospital	Average straight distance between the nearest Internet hospital^2^ and the unit’s geometric center (m).
	Density_Roadnet	Density of roadnet within the unit (km^−1^).
Demander	Num_Pop	Number of permanent residents in the unit.
	Ratio_Senior	Proportion of the people over 60 and above (or between 15–59) in the unit.
	Ratio_Youth	
	Income	Average housing price in the unit (Yuan).

^1^General hospital refers to a medical establishment providing multi-department diagnosis and treatment, while also supporting education, research, and preventive healthcare.

^2^Internet hospital is a platform that supplies multidisciplinary digital medical services, typically established by tertiary Grade-A hospitals.

#### 3.5.2 Assessment of explanatory factors using Geodetector

To assess the explanatory power of factors associated with the spatial heterogeneity of potential accessibility, this study employs Geodetector. The method is based on the premise that, when a factor is spatially associated with an outcome, their spatial distributions should exhibit similar stratified patterns. As the method requires categorical inputs, continuous variables were discretized by selecting the classification method and number of strata that minimized within-stratum variance and maximized between-stratum differences. The optimal specifications were determined through grid search using *R.*

The factor detector measures the individual explanatory power of each factor (Equation 20), with the q -value indicating the proportion of spatial variance associated with its stratification. The interaction detector evaluates the joint explanatory power of factor pairs, $q(X_{1}\cap X_{2})\;$ (Equation 21), by comparing the combined q -value with those of the individual factors.


$$q=1-\frac{\sum_{h=1}^{L}N_{h}\sigma_{h}^{2}}{N\sigma^{2}}$$ (20)

where q∈[0,1] denotes explanatory power; L is the number of strata; $N_{h}$ , N are sample sizes in stratum *h* and the whole region, respectively; and $\sigma_{h}^{2}$ , $\sigma^{2}$ are the variances of the dependent variable in the corresponding areas.


$$q(X_{1}\cap X_{2})=1-\frac{\sum_{h=1}^{L_{X_{1}}}\sum_{k=1}^{L_{X_{2}}}N_{\mathrm{hk}}\sigma_{\mathrm{hk}}^{2}}{N\sigma^{2}}$$ (21)

Where $\;L_{X_{1}}$ , $L_{X_{2}}$ are stratum counts of factors $X_{1}$ and $X_{2}$ ; $N_{\mathrm{hk}}$ is the sample size in the intersection of stratum h of $X_{1}$ and stratum k of $\;X_{2}$ ; $\sigma_{\mathrm{hk}}^{2}\;$ is the variance of the dependent variable in this intersection.

## 4 Results

### 4.1 Identification of groups with on-demand delivery advantage

#### 4.1.1 Demand group classification and validity assessment

**Figure 4:**
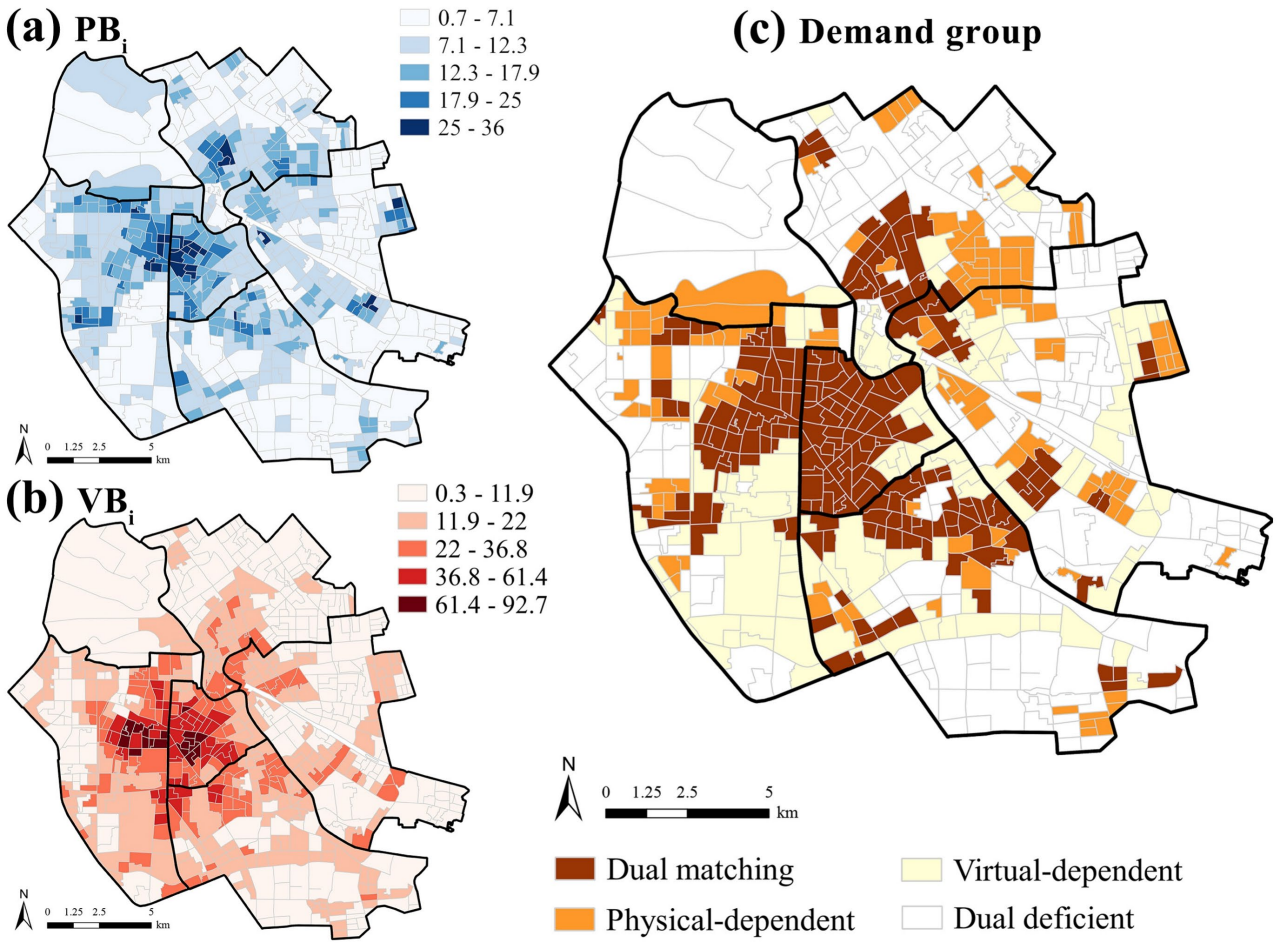
Demand group classification based on $PB_{i}$ and $VB_{i.}$

As shown in Figure 4, both $PB_{i}$ and $VB_{i}$ vary considerably across the 647 community units, with coefficients of variation of 0.59 and 0.83. This indicates substantial intra-urban heterogeneity in digital barriers. Based on the two indicators, 207, 117, 117, and 206 communities were classified as dual-matching, physical-dependent, virtual-dependent, and dual-deficient, respectively.

Table 5 presents the results of one-way ANOVA results. Significant group differences are observed in potential accessibility of in-store and delivery channels, and their relative difference (*p* < 0.05), supporting the validity of grouping approach in this study.

**Table 5:** Result of F-test.

Potential CPS accessibility	F	*p*-value
In-store channel	72.56	<0.05*
On-demand delivery channel	3.41	<0.05*
Relative difference between two channels	59.41	<0.05*

*statistical significance.

*F*-value is substantially higher for in-store potential accessibility (72.56) than for delivery-based potential accessibility (3.41), indicating stronger between-group differentiation in the in-store channel. Thus, modeled delivery-based service opportunities vary less across barrier groups, although this does not by itself demonstrate distributional equity. The high F-value for the relative accessibility difference (59.41) further indicates substantial group variation in the channel offering the stronger potential accessibility advantage.

#### 4.1.2 Potenital accessibility across two channels and their relative difference

**Figure 5:**
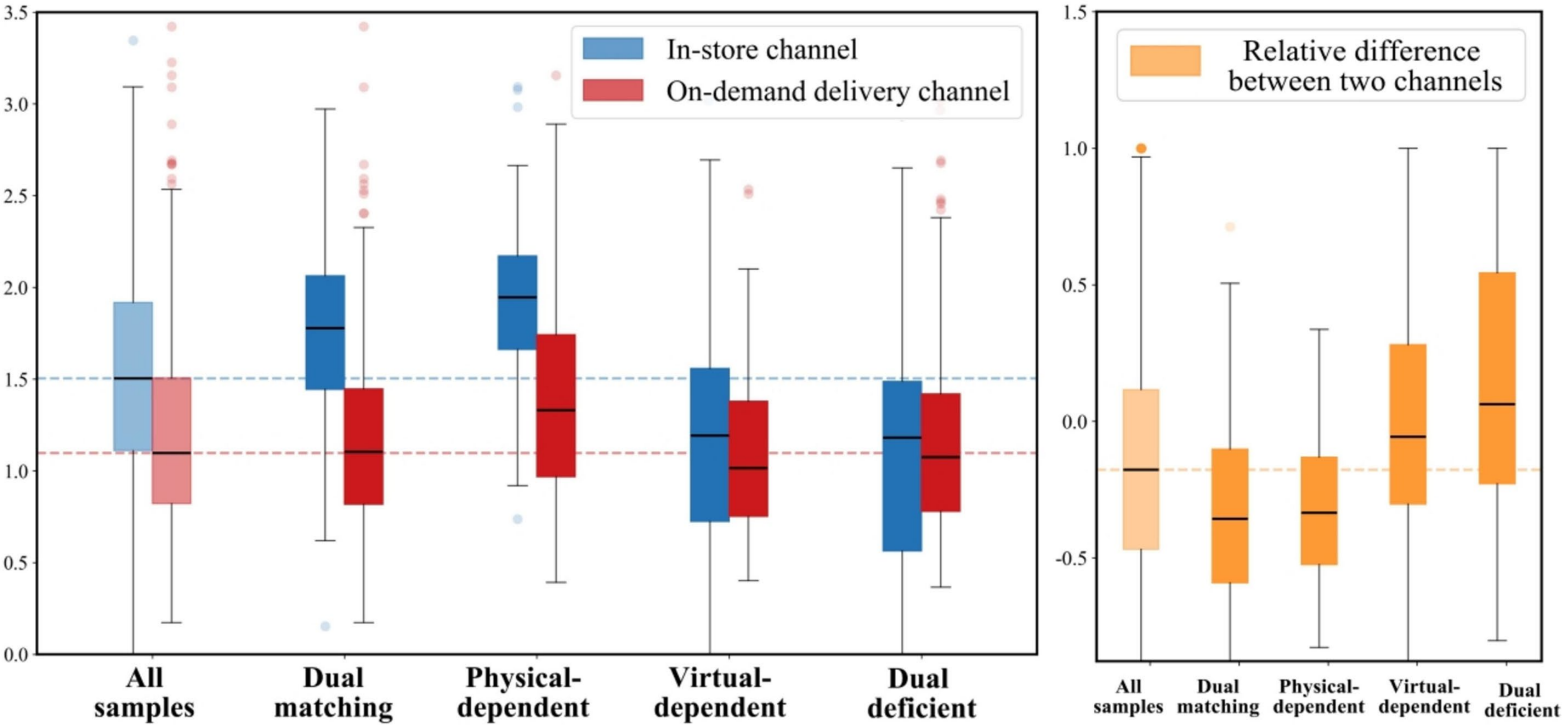
Accessibility across two channels and their relative difference. Dashed lines represent the corresponding median values for all samples.

**Figure 6:**
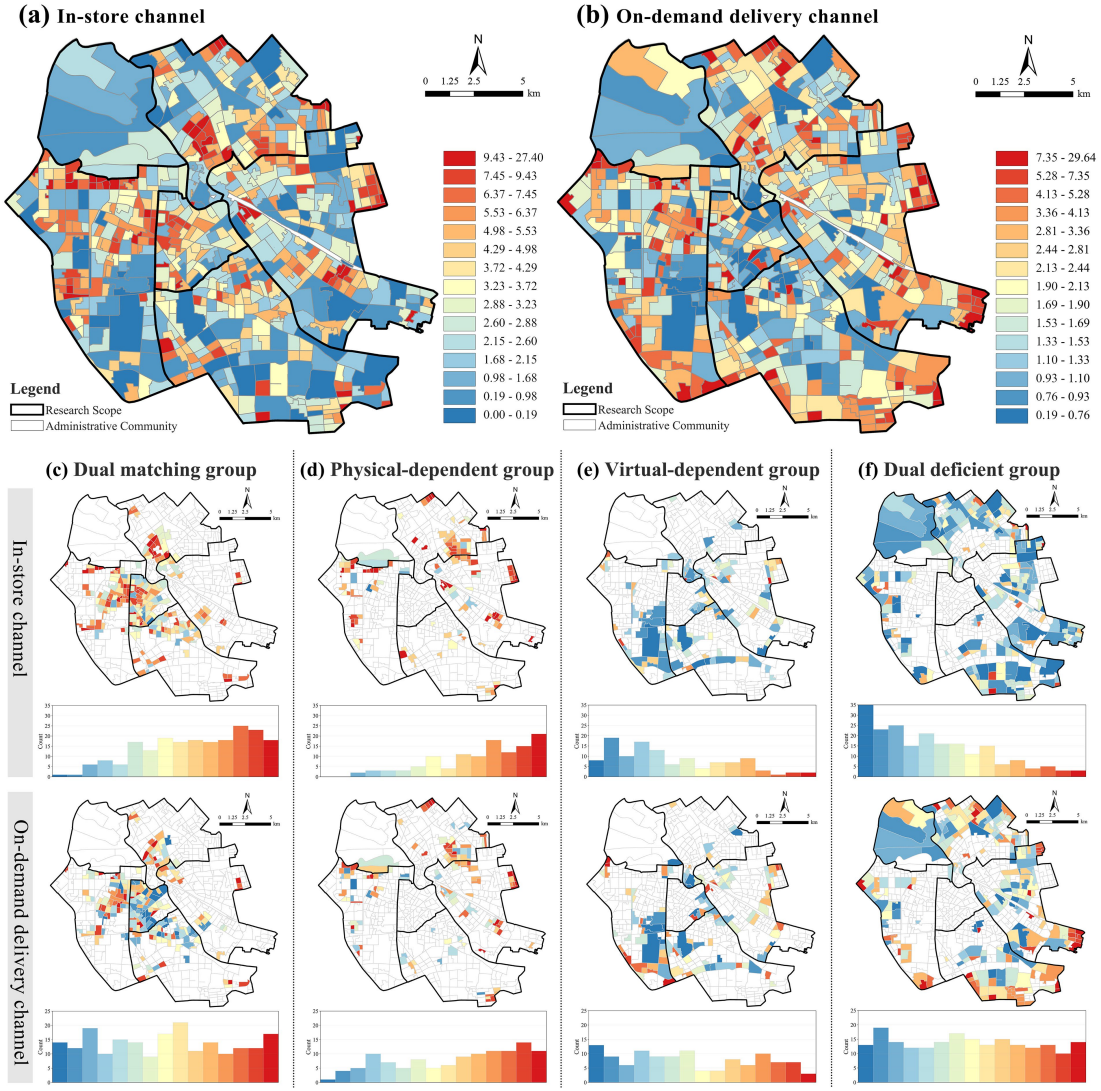
Spatial distribution of potential CPS accessibility.

Figure 5 compares in-store and delivery-based potential accessibility and their relative difference across demand groups, using all community units as the reference. Figure 6 shows their spatial distributions.

In terms of opportunity scale, delivery-based potential accessibility has lower medians and interquartile ranges (IQRs) than in-store potential accessibility across all groups. For the full sample, the median is 1.50 for in-store dispensing and 1.10 for on-demand delivery. The dual-matching and physical-dependent groups show higher median in-store accessibility (1.78 and 1.95) than delivery-based accessibility (1.10 and 1.33). For the virtual-dependent and dual-deficient groups, the IQRs of the two channels are broadly comparable, but delivery shows no overall scale advantage. Thus, the modeled volume of potential service opportunities provided by on-demand delivery remains lower than that provided by in-store dispensing.

The relative difference reveals distinct channel advantages across groups. The dual-matching and physical-dependent groups have negative median values, indicating higher potential accessibility through in-store dispensing. By contrast, the virtual-dependent and dual-deficient groups show slightly positive medians, and values from the median to the third quartile favor on-demand delivery. This pattern indicates that delivery provides a relative potential accessibility advantage for some communities facing high geographic barriers, although it does not represent actual channel preference or realized service use.

Spatially, the two channels exhibit contrasting patterns. High in-store potential accessibility is concentrated in the urban core, forming a center-to-periphery decline. Higher delivery-based potential accessibility is more common in peripheral areas, while central areas generally show lower values. These patterns reflect the different spatial organization of potential service opportunities across the two channels.

#### 4.1.3 Equity of potential accessibility

**Figure 7:**
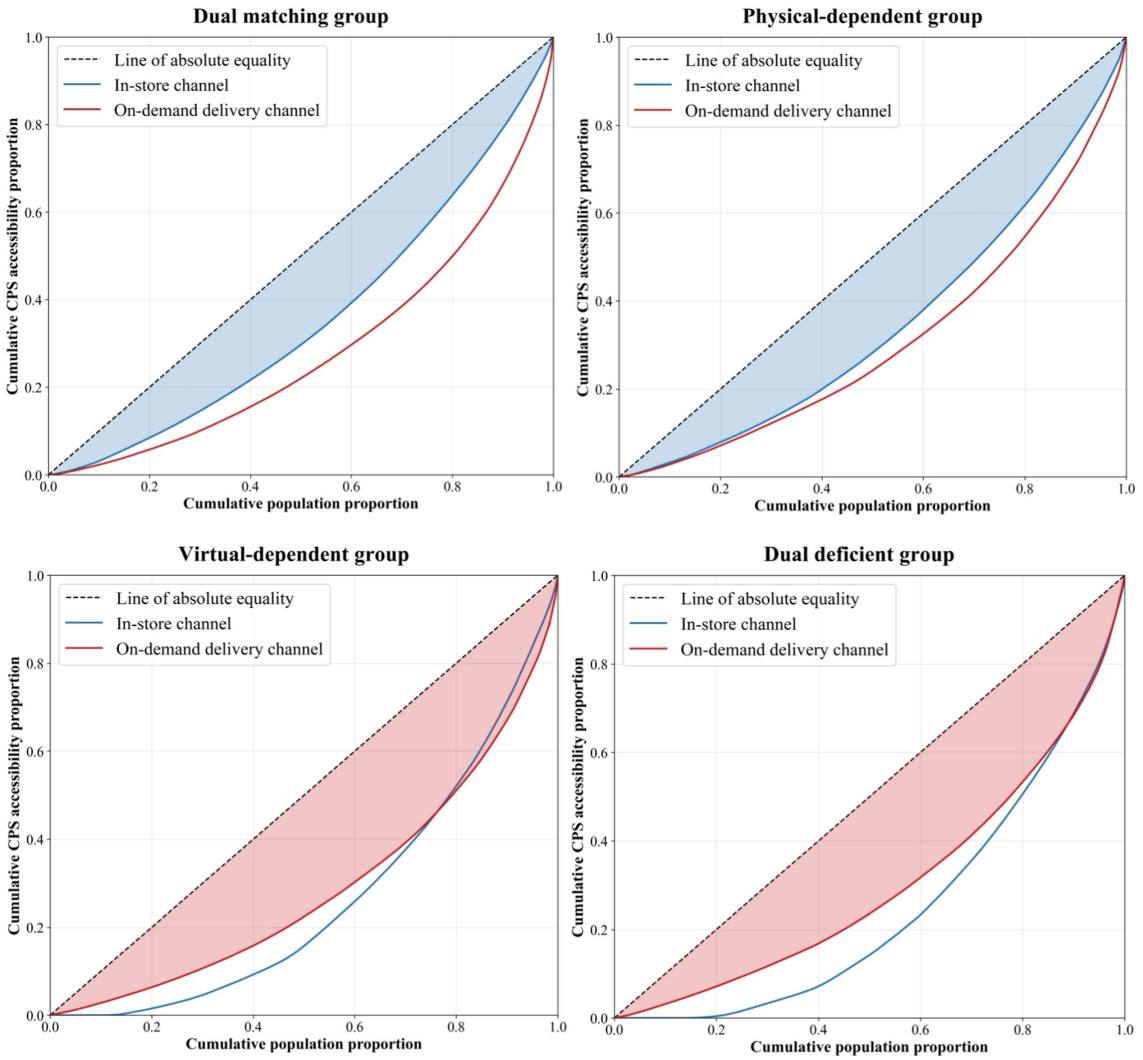
Lorenz curve of potential CPS accessibility.

Table 6 and Figure 7 present the Lorenz curves and Gini coefficients for in-store and delivery-based potential accessibility across demand groups. For the full sample, the Gini coefficient for the delivery channel is 0.08 lower than that for in-store dispensing, indicating a more even distribution of modeled potential service opportunities.

**Table 6:** Gini coefficients of potential CPS accessibility.

Potential CPS accessibility	All samples	Dual matching	Physical-dependent	Virtual-dependent	Dual deficient
In-store channel	0.47	0.28	0.31	0.47	0.50
On-demand delivery channel	0.39	0.29	0.29	0.39	0.38
Difference	0.08	−0.01	0.02	0.08	0.12

This difference varies across groups. In the dual-matching and physical-dependent groups, the two channels show similar levels of distributional equity. By contrast, in the virtual-dependent and dual-deficient groups, the Gini coefficients for in-store potential accessibility exceed 0.4, whereas those for delivery-based accessibility are lower by 0.08 and 0.12 and fall below 0.4. Thus, on-demand delivery is associated with a more equitable distribution of potential CPS opportunities in groups facing high geographic barriers.

### 4.2 Analysis of factors associated with CPS accessibility

To better understand the potential contribution of on-demand delivery to CPS accessibility, this study examines the explanatory power of relevant factors for the virtual-dependent and dual deficient groups. Using factors associated with in-store potential accessibility as a reference, this section further compares their explanatory power for the spatial heterogeneity of delivery-based accessibility.

#### 4.2.1 Virtual-dependent group

This group faces high geographic but low digital barriers, and on-demand delivery shows a potential accessibility advantage. Factors associated with potential CPS accessibility differ substantially between the in-store and delivery channels.

##### 4.2.1.1 Independent explanatory power

**Figure 8:**
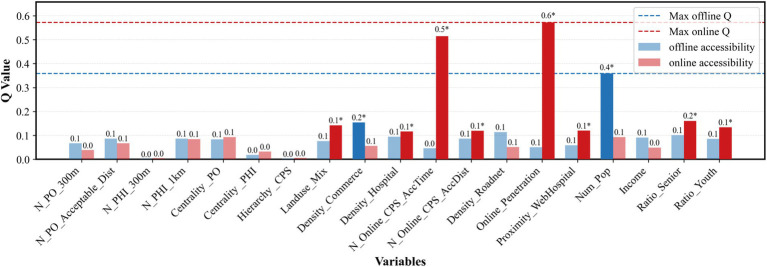
Independent explanatory power on CPS potential accessibility.

Figure 8 presents the explanatory power of individual factors for potential accessibility in the two channels. Overall, the factors show greater explanatory power for delivery-based accessibility.

For in-store accessibility, demand-side factors are more prominent, with demand scale showing the highest explanatory power (*q* = 0.36). For delivery-based accessibility, supply-side factors show stronger associations. CPS online penetration (*q* = 0.57) and the number of online suppliers within acceptable delivery time (*q* = 0.52) have the highest explanatory power, highlighting the importance of supply scale. Proportion of people over 60 (*q* = 0.16) shows a weaker but notable association.

##### 4.2.1.2 Interaction explanatory power

**Figure 9:**
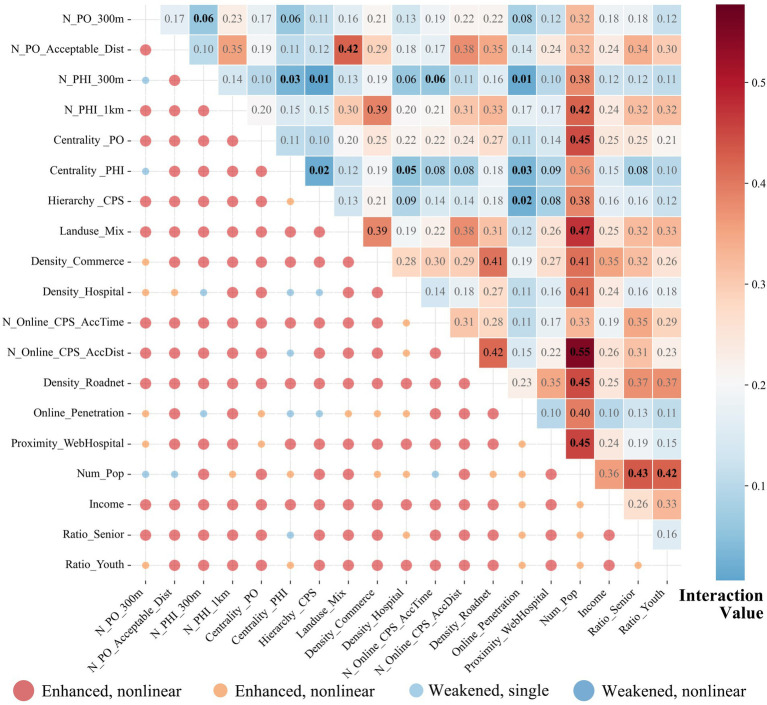
Interaction explanatory power on in-store accessibility.

**Figure 10:**
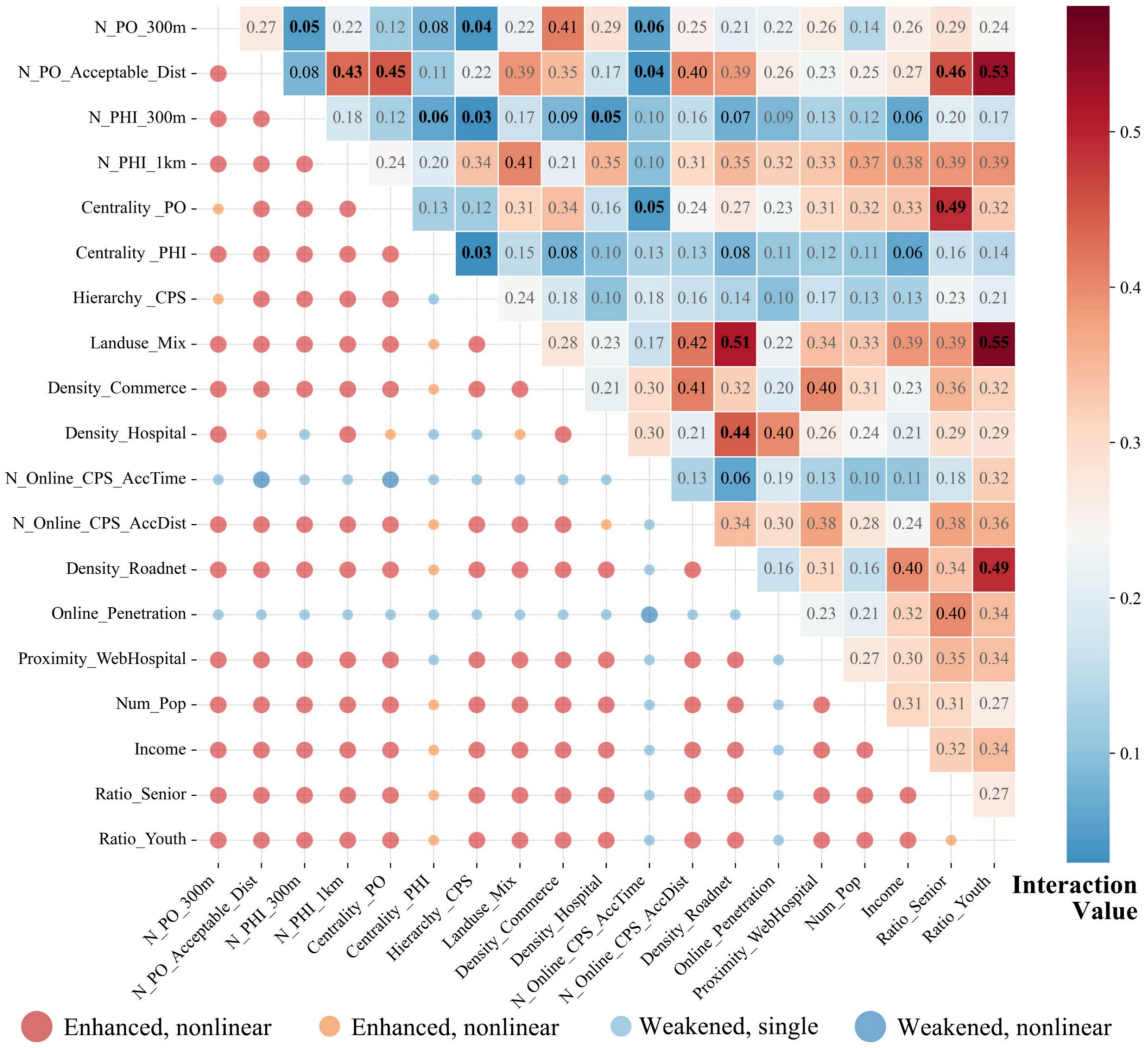
Interaction explanatory power on delivery-based accessibility.

Figures 9, 10 show that factor interactions generally have greater explanatory power than individual factors, although the leading factors and interaction patterns differ between channels.

For in-store accessibility, demand scale shows the strongest interactions with other factors, particularly the number of online suppliers, land-use mix, and PO centrality (max *q* = 0.55), indicating that the traditional channel is more strongly associated with the synergy between population concentration and facility allocation than with either factor alone.

For delivery-based accessibility, age structure shows the strongest interactions with spatial factors, particularly through the proportions of young and older residents, still dominated by nonlinear enhancement. By contrast, CPS online penetration and the number of online suppliers generally show lower joint explanatory power when combined with other factors. These results indicate distinct spatial association patterns between the two channels rather than causal mechanisms.

#### 4.2.2 Dual deficient group

This group faces both high geographic and digital barriers and shows unfavorable potential accessibility in both channels. The factors associated with potential CPS accessibility also differ substantially between the in-store and delivery channels.

##### 4.2.2.1 Independent explanatory power

**Figure 11:**
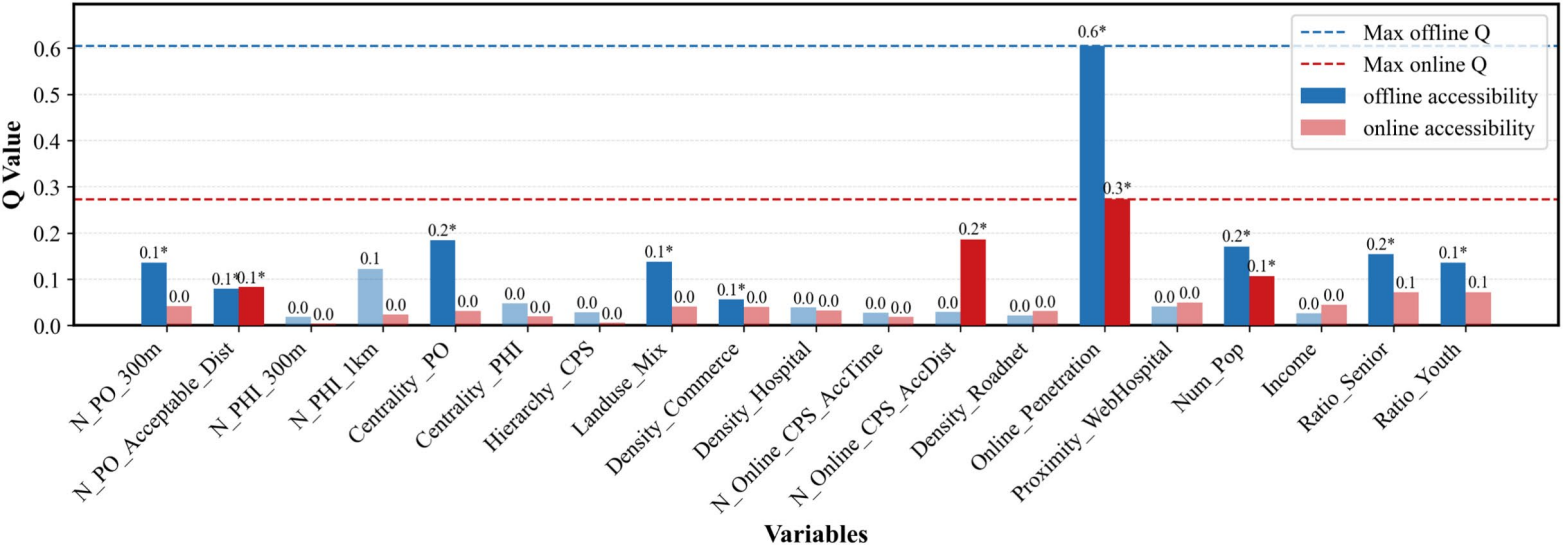
Independent explanatory power on CPS potential accessibility.

Figure 11 presents the explanatory power of individual factors for potential accessibility in the two channels. Only a few factors show substantial explanatory power, while most are weakly associated with spatial heterogeneity.

For in-store accessibility, CPS online penetration shows the highest explanatory power (*q* = 0.60), far exceeding other factors. This may reflect the role of digital platforms in increasing the visibility of pharmacy locations and service information, even for in-store access. For delivery-based accessibility, overall explanatory power is lower, with CPS online penetration again showing the highest value (*q* = 0.27).

##### 4.2.2.2 Interaction explanatory power

**Figure 12:**
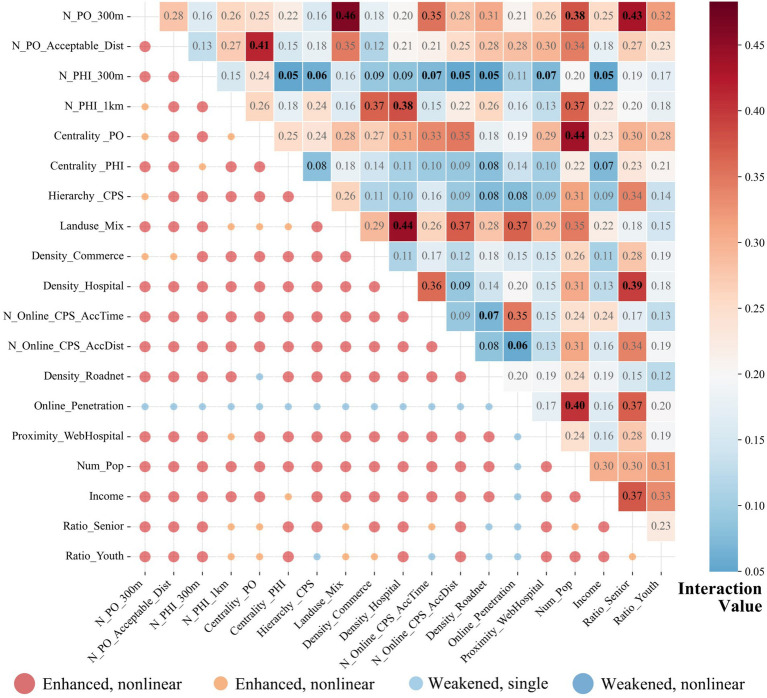
Interaction explanatory power on in-store accessibility.

**Figure 13:**
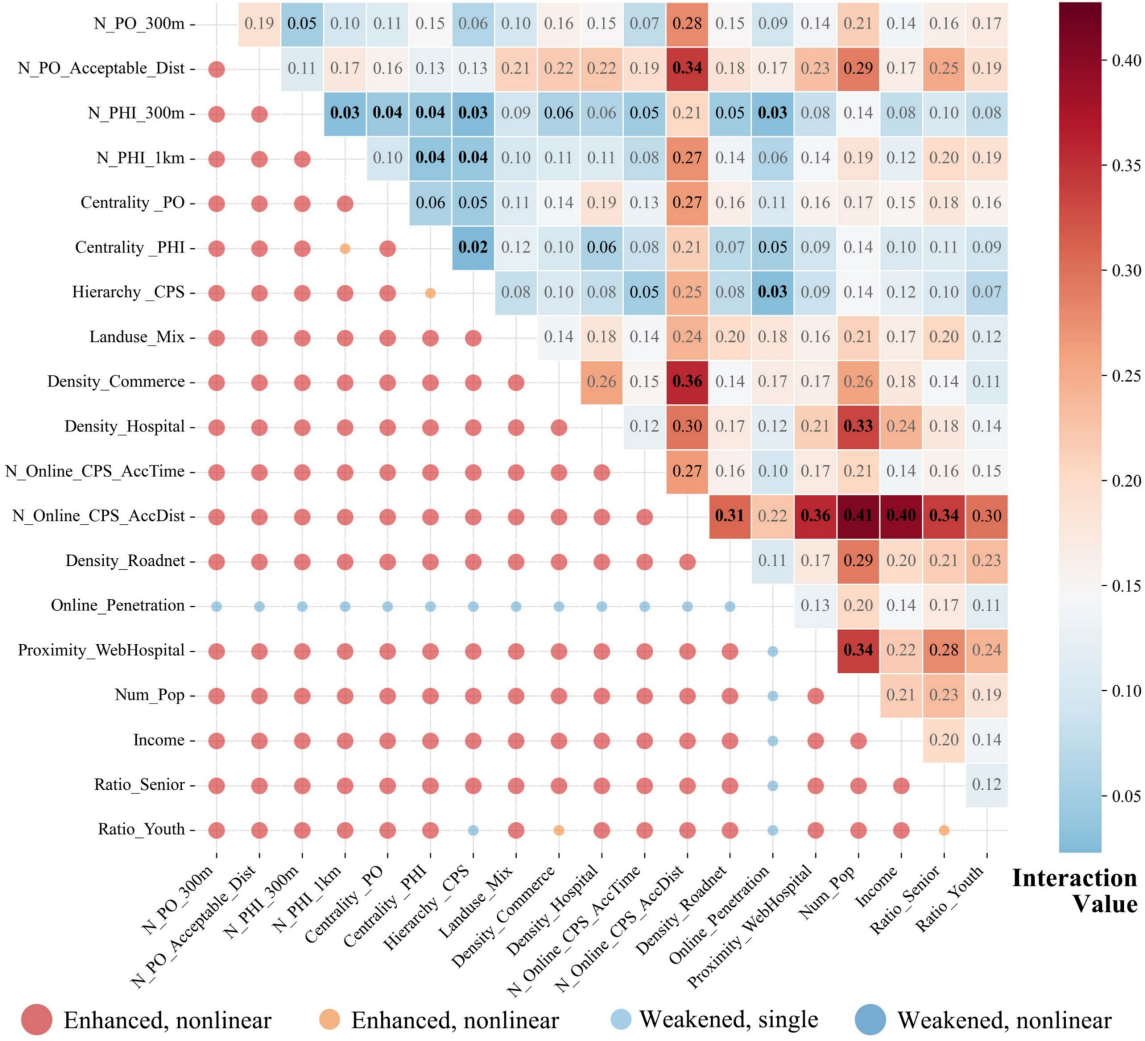
Interaction in-store on delivery-based accessibility.

Figures 12, 13 show that most factor interactions exhibit nonlinear enhancement, although only a few pairs have relatively high explanatory power for delivery-based accessibility.

For in-store accessibility, the strongest interactions involve pharmacy establishments and population structure: the number of POs within 300 m range interacts most strongly with land-use mix (*q* = 0.46) and the older population share (*q* = 0.43). Demand scale also interacts stably with PO location centrality (*q* = 0.44) and online penetration rate of CPS (*q* = 0.40).

For delivery-based accessibility, the number of online suppliers shows the strongest interactions with demand scale (*q* = 0.41) and consumption level (*q* = 0.40). This further indicates that population concentration and economic capacity are key constraints for this doubly vulnerable group in using on-demand delivery.

## 5 Discussion

### 5.1 Theoretical contributions

Responding to the digitalization of pharmaceutical services, this study extends the conventional 2SFCA model to measure dual-channel potential CPS accessibility by integrating objective spatial conditions with survey-derived perceptions. First, supply capacity is quantified using a multidimensional, preference-weighted framework covering approachability, availability, affordability, and appropriateness, rather than relying solely on facility size or quantity (15, 53). Channel-specific indicators are adopted to reflect differences between in-store dispensing and on-demand delivery. Second, modeled supplier-selection probability is introduced to reduce the assumption that all reachable suppliers are equally selected. Travel time represents competition among in-store suppliers, while platform ranking captures relative visibility and competitiveness in the delivery channel (45, 65). These refinements support a behaviorally informed assessment of potential accessibility by combining objective supplier attributes with residents’ stated preferences (25, 43).

### 5.2 Practical implications

CPS allocation is a major concern in urban planning and public health. As on-demand delivery becomes an important complement to in-store dispensing, overlooking group heterogeneity may reinforce accessibility disparities associated with geographic and digital divides (17, 23). Accordingly, this study proposes the following implications.

First, the relative contribution of on-demand delivery to potential CPS accessibility and equity varies across demand groups. For groups facing geographic or dual barriers, delivery does not provide a larger overall volume of potential service opportunities than in-store dispensing, but it shows a relative accessibility advantage and a more equitable spatial distribution. This supports the targeted use of delivery services to improve potential CPS accessibility (39). Geodetector analysis further shows that factor interactions generally have greater explanatory power than individual factors and predominantly exhibit nonlinear enhancement. The resulting group-specific implications are as follows.

For communities with low geographic barriers, in-store dispensing already provides relatively high potential accessibility and an equitable distribution of service opportunities. Delivery services may therefore serve mainly as a supplementary option, particularly for urgent or nighttime needs (10, 15). This strategy is relevant to compact urban areas with relatively well-developed offline service networks (63).

For communities with low digital but high geographic barriers, delivery-based services should receive greater planning attention. Governments may subsidize delivery fees for chronic or urgent medications and gradually integrate eligible online purchases into medical-insurance and primary-care systems (64). Platforms can reduce entry costs and commissions, while public subsidies can encourage more pharmacies to provide delivery services. These measures are particularly applicable to suburban and new-town areas with adequate digital infrastructure but limited physical facilities.

Communities facing both geographic and digital barriers require coordinated offline and online interventions, particularly in remote or socioeconomically disadvantaged areas (55, 56). Primary healthcare institutions can provide digital-skills assistance for older adults, while front warehouses, expanded online supply, and delivery subsidies can improve the availability of potential service opportunities in underserved areas. Such combined measures may support more equitable pharmaceutical-service planning where infrastructure and service capacity are limited.

## 6 Conclusion and limitations

In the digital era, in-store dispensing and on-demand delivery jointly shape potential access to CPS, making group-specific accessibility and equity an important concern for public health and spatial planning. To address insufficient consideration of channel-specific barriers, limited measurement of potential accessibility, and inadequate attention to factor interactions, this study extends the 2SFCA model by integrating objective spatial conditions with survey-derived perceptions. Community units are classified into four groups according to geographic and digital barriers. Potential accessibility and its distributional equity are then compared across channels and groups, followed by Geodetector analysis of the individual and joint explanatory power of factors associated with spatial heterogeneity.

Key findings include: (1) the relative contribution of on-demand delivery varies across groups. In-store dispensing provides higher potential accessibility for groups with low geographic barriers, whereas delivery shows a relative accessibility advantage and a more equitable distribution of potential service opportunities in the virtual-dependent and dual-deficient groups. (2) Factor interactions generally show greater explanatory power than individual factors and predominantly exhibit nonlinear enhancement. The factors associated with spatial heterogeneity also differ between channels, with delivery-based potential accessibility more closely related to the joint distribution of online supply, age structure, and socioeconomic characteristics.

Beyond these findings, several limitations should be noted. (1) Data limitations restrict the representation of demand-side heterogeneity. Variables such as income, gender, health status, medication needs and digital literacy across all age groups should be incorporated in future research to better characterize potential participation in each channel (57). (2) Data precision is limited by model assumptions and temporal mismatch. A common time-decay function is applied across medication needs, although urgent purchases may be more time-sensitive than routine refills, for which price, insurance eligibility, and supplier reliability may matter more. Future studies should estimate demand-specific impedance functions. In addition, population size and age structure came from the 2020 Seventh National Population Census, which may not fully reflect later demographic changes. (3) The study focuses on central urban areas and excludes rural settings, where longer delivery distances, weaker logistics, and limited digital infrastructure may create additional barriers (16). The findings should therefore be tested in other spatial contexts.

## Data Availability

The raw data supporting the conclusions of this article will be made available by the authors, without undue reservation.
