# Supplementary material for: How does on-demand delivery impact the accessibility and equity of community pharmacies? Perspective from groups with heterogeneous geographic-digital barriers
Source: Front Public Health. 2026 Jul 29;14:1872047. doi: 10.3389/fpubh.2026.1872047 (PMC13461892; doi:10.3389/fpubh.2026.1872047)
Supplement: Supplementary file 1 [file Supplementary_file_1.DOCX]

Supplementary Material

# Supplementary Note 1.

**Supplementary Table 1** Stratified sampling by age structure

| **Age group (Years old)** | **Sample size** | **Number of valid questionnaires** |
| --- | --- | --- |
| ≤ 19 | 110 | 90 |
| 20–29 | 75 | 64 |
| 30–39 | 110 | 93 |
| 40–49 | 85 | 70 |
| 50–59 | 85 | 76 |
| ≥ 60 | 135 | 108 |
| **Total** | **600** | **501** |

**Supplementary Note 2. Survey template on pharmaceutical acquisition behavior**

# Dear Respondents,

# Thank you for participating in our survey, which aims to understand your daily consumption behavior regarding online and offline medicine purchases to enhance community pharmaceutical services. This survey is for academic research purposes only, and all your responses will remain strictly confidential.

# Q1. What is your age?

# □19 and below □20 –29 □30 –39 □40 –49 □50 –59 □60 and above

# Q2. In which district do you reside?

# □Heping District □Hebei District □Hedong District

# □Hexi District □Nankai District □Hongqiao District

# Q3. When purchasing medicine from a primary healthcare institution (PHI), what is the maximum walking time you are willing to accept?

# □5 minutes □10 minutes □15 minutes □20 minutes □25 minutes

# Q4. When purchasing medicine from a pharmaceutical outlet (PO), what is the maximum walking time you are willing to accept?

# □5 minutes □10 minutes □15 minutes □20 minutes □25 minutes

# Q5. Out of every 10 offline medicine purchases, how many times do you choose a pharmaceutical outlet (PO) versus a primary healthcare institution (PHI)?

# (Please fill in the blanks with numbers from 0 to 10)

# Pharmaceutical Outlet (PO): ____ times

# Primary Healthcare Institution (PHI): ____ times

# Q6. When purchasing medicine through online delivery platforms, what is the maximum delivery time you can accept?

# □29 minutes or less □30–39 minutes □40–49 minutes □50–59 minutes □61 minutes or more

# Q7. When purchasing medicine online, which ranking positions do you tend to select from the app’s recommendations?

# □Top 1–3 (Upper half of the 1st page)

# □Top 4–6 (Lower half of the 1st page)

# □Top 7–9 (Upper half of the 2nd page)

# □Top 10–12 (Lower half of the 2nd page)

# □13th position and beyond (3rd page and beyond)

# Q8. When choosing an online community pharmaceutical service provider, which of the following information is most important to you? Please rank them in order of importance (1–6, where 1 = most important, 6 = least important).

# ____ Meituan-dedicated delivery

# ____ Store rating

____ 24-hour operation

# ____ Delivery fee

# ____ Minimum order price

# ____ Coupon number

# Q9. When choosing a PO, which of the following information is most important to you? Please rank them in order of importance (1–4, where 1 = most important, 4 = least important).

# ____ Proximity

# ____ Walking convenience

# ____ Medical insurance qualification

____ 24-hour operation

# Q10. When choosing a PHI, which of the following information is most important to you? Please rank them in order of importance (1–4, where 1 = most important, 4 = least important).

# ____ Proximity

# ____ Walking convenience

# ____ Medical insurance qualification

____ 24-hour operation

# Supplementary Note 3. Weights of multidimensional supply capacity for CPS

Based on survey data regarding pharmacy consumption behavior, Supplementary Figure 1 summarizes residents’ importance rankings for each dimension of supply capacity. Table 2 further calculates the weight of each dimension in the supply capacity based on its frequency of selection across different ranking positions.

In the in‑store channel, the frequencies of obtaining medicines from pharmaceutical outlets (PO) and primary health institutions (PHI) are similar, with PO slightly higher (52.33%). Further analysis reveals clear differences in residents’ preference for dimensions between the two facility types: (1) For PO, spatial proximity rated as the most important factor by the highest proportion of respondents (Roubin et al., 2025), followed closely by medical insurance qualification, the factor ranked first by the most participants, highlighting the role of economic and payment security considerations (Wang et al., 2025). Walking convenience and 24-hour operation are regarded as less critical. (2) For PHI, medical insurance qualification and spatial proximity are nearly equally important, with the former still viewed as the most decisive factor, underscoring its significance for using physical facilities. Residents also value walking convenience more for PHI than for PO.

In the delivery channel, store rating is the most valued dimension, followed by delivery fee and minimum order price, indicating that service reputation and cost are key decision factors for online medicine purchases. The high importance placed on 24-hour operation reflects a clear demand for temporal flexibility in pharmaceutical services, especially for after‑hours emergency access.

**Supplementary Fig.1.** Residents’ importance rankings for each dimension of supply capacity

# Supplementary Note 4. Fitting of the time decay function

Figure 2 presents residents’ maximum acceptable durations for in‑store dispensing (maximum walking time) and on‑demand delivery (maximum delivery time), which are used to define the spatial impedance of pharmaceutical access. Based on the distribution of selection probabilities over time, the specific form of the time decay function is fitted.

For the in‑store channel: (1) For PO, the weighted‑average maximum acceptable walking time $t_{0}^{P}$ is 11.7 minutes. The decline in selection probability with increasing walking time follows a Gaussian form (*R^2^* = 0.95); therefore, a Gaussian time‑decay function $g\left( t_{kj} \right)$ (Equation 1) is adopted, with the value dropping to 0 beyond the threshold. (2) For PHI, the maximum acceptable walking time $t_{0}^{P}$ is 13.6 minutes, and its decay also conforms to a Gaussian pattern (*R^2^* = 0.86), reflecting consistent travel behavior toward physical medical facilities.

For the delivery channel, the maximum acceptable delivery time $t_{0}^{V}$ is 43 minutes, significantly longer than the walking thresholds for in‑store dispensing. Its decay is likewise well described by a Gaussian function (*R^2^* = 0.76), and the same Gaussian form of $g\left( t_{kj} \right)$ is applied accordingly.

**Supplementary Fig.2.** Residents’ maximum acceptable durations for in‑store and delivery channel

$$\boldsymbol{g}\left( \boldsymbol{t}_{\boldsymbol{kj}} \right)\boldsymbol{=}\frac{\boldsymbol{e}^{{\boldsymbol{-}\left( {\boldsymbol{t}_{\boldsymbol{kj}}}/{\boldsymbol{t}_{\boldsymbol{0}}} \right)^{\boldsymbol{2}}}/\boldsymbol{2}}\boldsymbol{-}\boldsymbol{e}^{\boldsymbol{-1}/\boldsymbol{2}}}{\boldsymbol{1-}\boldsymbol{e}^{\boldsymbol{-1}/\boldsymbol{2}}}\boldsymbol{,}\boldsymbol{t}_{\boldsymbol{kj}}\boldsymbol{\leq}\boldsymbol{t}_{\boldsymbol{0}}$$

(1)

# Supplementary Note 5. Fitting of supplier selection probability

For the in‑store channel, supply competition is primarily determined by travel time. Thus, the Gaussian decay function based on travel time, as derived in Section 3.2, is adopted directly to represent the decay of supplier *j* under travel‑time competition *t* (Equation 2).

For the delivery channel, Figure 3 presents the scatter distribution of residents’ supplier selection probability against platform ranking position r. The data show a clear exponential decay trend (*R²* = 0.92). Therefore, the decay function for any on‑demand supplier *j* at ranking position *r* is expressed as Equation 3.

$$\boldsymbol{h}\left( \boldsymbol{t}_{\boldsymbol{j}} \right)\boldsymbol{=}\frac{\boldsymbol{e}^{{\boldsymbol{-}\left( {\boldsymbol{t}_{\boldsymbol{ij}}}/{\boldsymbol{t}_{\boldsymbol{0}}} \right)^{\boldsymbol{2}}}/\boldsymbol{2}}\boldsymbol{-}\boldsymbol{e}^{\boldsymbol{-1}/\boldsymbol{2}}}{\boldsymbol{1-}\boldsymbol{e}^{\boldsymbol{-1}/\boldsymbol{2}}}\boldsymbol{,}\boldsymbol{t}_{\boldsymbol{ij}}\boldsymbol{\leq}\boldsymbol{t}_{\boldsymbol{0}}$$

(2)

$$\boldsymbol{h}\left( \boldsymbol{r}_{\boldsymbol{j}} \right)\boldsymbol{=165.62}\boldsymbol{e}^{\boldsymbol{-0.445}\boldsymbol{r}_{\boldsymbol{j}}}$$

(3)

**Supplementary Fig.3.** Scatter distribution of supplier selection probability against platform ranking
